# Supplementary material for: Dissecting the Roles of Cuticular Wax in Plant Resistance to Shoot Dehydration and Low-Temperature Stress in Arabidopsis
Source: Int J Mol Sci. 2021 Feb 4;22(4):1554. doi: 10.3390/ijms22041554 (PMC7913816; doi:10.3390/ijms22041554)
Supplement: Supplementary file 1 [file ijms-22-01554-s001.pdf]

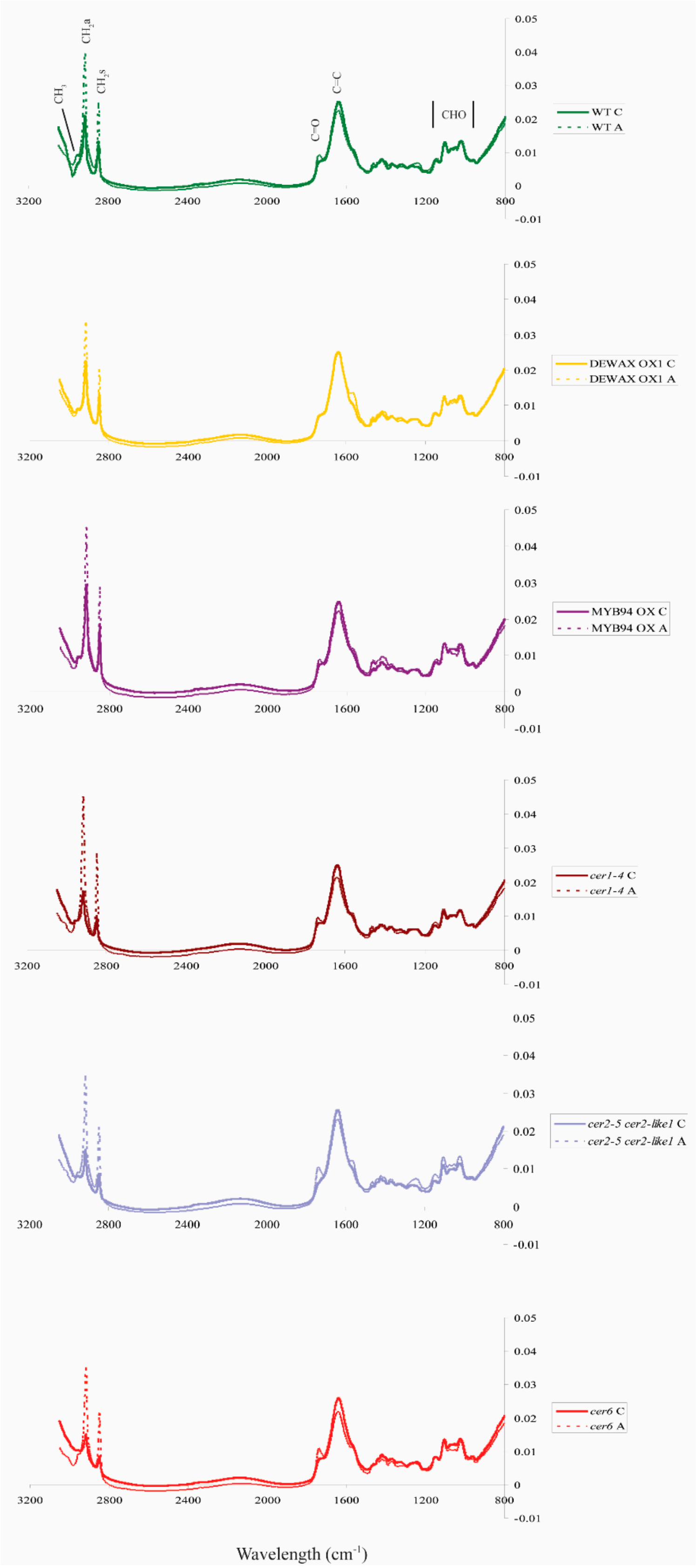

Figure S1. ATR-FTIR spectral profiles of rosette leaf adaxial surfaces under control (C; solid lines) and cold-acclimated (A; dotted lines) conditions for WT, DEWAX OX1, MYB94 OX, *cer1-4*, *cer2-5 cer2-like1*, *cer6* presented.

Table S1. Pearson correlation values between GC-MS and ATR-FTIR data.

	CH_3	CH_2a	CH_2s	CO	CC	RCH32s	RCO2s	RCC2s
C24.FA	0.88	0.63	0.47	0.80	−0.86	0.21	0.21	−0.44
C26.FA	0.91	0.82	0.69	0.59	−0.96	−0.04	−0.06	−0.66
C28.FA	0.64	0.98	0.99	−0.11	−0.85	−0.69	−0.73	−0.98
C30.FA	−0.08	0.57	0.71	−0.56	−0.27	−0.96	−0.85	−0.75
C32.FA	−0.74	−0.23	−0.04	−0.69	0.50	−0.60	−0.44	−0.03
C34.FA	−0.85	−0.51	−0.33	−0.63	0.72	−0.31	−0.20	0.28
C29.H	−0.31	0.38	0.53	−0.64	−0.04	−0.90	−0.76	−0.55
C31.H	−0.63	−0.02	0.15	−0.67	0.32	−0.72	−0.54	−0.19
C33.H	−0.66	−0.05	0.12	−0.68	0.35	−0.71	−0.53	−0.17
C35.H	−0.46	0.16	0.33	−0.61	0.13	−0.81	−0.63	−0.39
C26.OH	−0.95	−0.57	−0.40	−0.76	0.83	−0.32	−0.22	0.38
C28.OH	−0.92	−0.51	−0.33	−0.84	0.80	−0.40	−0.33	0.30
C29.OH	0.65	0.98	0.99	−0.10	−0.85	−0.68	−0.73	−0.98
C30.OH	−0.09	0.55	0.68	−0.61	−0.24	−0.95	−0.86	−0.72
C32.OH	−0.15	0.47	0.62	−0.55	−0.19	−0.91	−0.78	−0.67
C34.OH	−0.41	0.23	0.40	−0.58	0.06	−0.83	−0.65	−0.45

Table S2. ATR-FTIR spectroscopic data (partial) of cauline leaf adaxial cuticle surfaces generated under cold acclimation from WT, *cer1-4* and *cer3-6*.

	CH_3 stretching (2966-2950) (CH_3)	CH_2 asymmetric (2936-2894) (CH_2a)	CH_2 symmetric (2871-2826) (CH_2s)
**WT A**	0.16 ± 0.011	1.23 ± 0.05	0.51 ± 0.02
***cer1-4* A**	0.14 ± 0.002	1.36 ± 0.04	0.57 ± 0.02
***cer3-6* A**	0.122 ± 0.01* (p = 0.02; WT A vs. *cer3-6*A)	0.56 ± 0.08** (p = 0.003; WT A vs. *cer3-6*A)	0.186 ± 0.04** (p = 0.0008; WT A vs. *cer3-6*A)
